# Glycosphingolipids in Dementia: Insights from Mass Spectrometry and Systems Biology Approaches

**DOI:** 10.3390/biomedicines13122854

**Published:** 2025-11-22

**Authors:** Mirela Sarbu, Raluca Ica, Maria-Roxana Biricioiu, Liana Dehelean, Alina D. Zamfir

**Affiliations:** 1 National Institute for Research and Development in Electrochemistry and Condensed Matter, 300224 Timisoara, Romania; mirela.sarbu86@yahoo.co.uk (M.S.); raluca.ica@gmail.com (R.I.); maria.biricioiu99@e-uvt.ro (M.-R.B.); 2 Department of Mathematics-Informatics, Aurel Vlaicu University of Arad, 310130 Arad, Romania; 3 Department of Physics, West University of Timisoara, 300223 Timisoara, Romania; 4 Neuroscience Department, Faculty of Medicine, Victor Babeş University of Medicine and Pharmacy, 300041 Timisoara, Romania; lianadeh@umft.ro; 5 Department of Technical and Natural Sciences, Aurel Vlaicu University of Arad, 310130 Arad, Romania

**Keywords:** glycosphingolipids, dementia, mass spectrometry, systems biology approaches, biomarkers

## Abstract

This narrative literature review synthesizes recent evidence on glycosphingolipid (GSL) dysregulation in dementia, emphasizing discoveries enabled by mass spectrometry (MS) and systems biology. Focusing on the research published within the last decade, we selected studies that are relevant to GSL alterations in dementia and notable for their methodological advances. The findings were conceptually integrated to emphasize key molecular, analytical, and systems-level aspects across the major dementia types. The results from MS-based glycolipidomics in Alzheimer’s disease, dementia with Lewy bodies, frontotemporal dementia, Parkinson’s disease dementia, and Huntington’s disease consistently indicate altered GSL metabolism and shared molecular vulnerabilities in neuronal lipid regulation. At the same time, distinct GSL signatures differentiate individual dementias, reflecting the disease-specific mechanisms of neurodegeneration. The literature also reveals that recent advances in high-resolution MS and integrative analytical workflows have shifted GSL research from descriptive to mechanistic, facilitating the detailed mapping of species linked to neuroinflammation, protein aggregation, and synaptic dysfunction. Systems-level analyses combining MS data with other omics approaches increasingly depict GSLs as active regulators of neuronal function rather than inert membrane components. At the same time, emerging trends position GSLs as promising early biomarkers and potential therapeutic targets, while the growing use of artificial intelligence in MS data analysis is accelerating the detection of their subtle patterns, improving cross-disease comparisons. Together, these results reinforce the major role of MS-based platforms in discovering dementia-associated GSLs, identifying therapeutic targets, and influencing future strategies for diagnosis and treatment.

## 1. Introduction

Dementia encompasses a heterogeneous group of progressive neurodegenerative disorders that together represent one of the most critical global health challenges of the twenty-first century. Defined by cognitive decline severe enough to interfere with independent living, dementia is not a single disease but rather a syndrome with multiple etiologies and pathological substrates [1].

Dementia prevalence is rising inexorably. According to the most recent epidemiological estimates by the World Health Organization, more than 55 million people live at present with dementia worldwide, and projections suggest that this figure will exceed 135 million by 2050, due to increased life expectancy and population aging. The consequences extend beyond patients to caregivers, healthcare systems, and societies at large, with deep medical, social, and economic implications.

**Figure 1:**
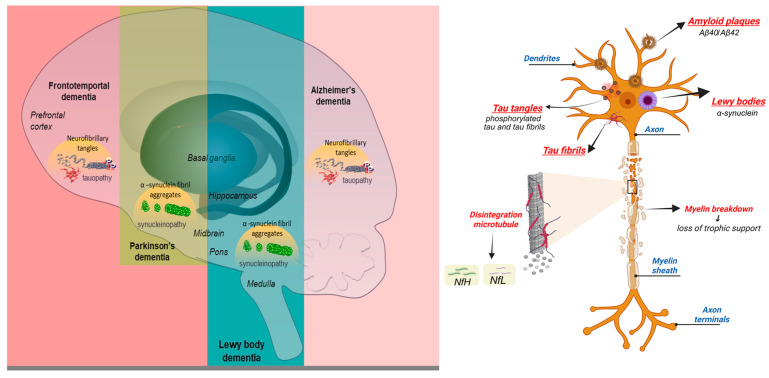
Molecular basis and hallmarks of major dementia types (schematic of neuron created in BioRender. Sarbu, M. (2025), https://BioRender.com/9gcz0t7, accessed on 5 October 2025).

Despite the intense research efforts over the past few decades, the development of effective therapies has been hampered by the remarkable clinical and molecular heterogeneity of dementia syndromes [2,3], such as Alzheimer’s disease (AD), dementia with Lewy body (DLB), frontotemporal dementia (FTD), Parkinson’s disease dementia (PDD), Huntington’s disease (HD), and mixed dementia. Each exhibits distinct clinical and neuropathological features yet converges on shared mechanisms such as synaptic dysfunction, protein aggregation, mitochondrial impairment, oxidative stress, and neuroinflammation (Table 1) [4,5,6,7,8,9,10,11,12,13,14,15,16]. The molecular basis of the major dementia types, highlighting both shared pathways and disease-specific features, isillustrated in Figure 1. Progressively, the dysregulation of lipid metabolism has emerged as a critical contributor to these processes [17], pointing toward a dimension of dementia biology that has been neglected to some extent.

Lipids constitute nearly half of the brain’s dry weight and are essential for neuronal membrane structure, synaptic signaling, vesicular transport, and myelin stability [17,18]. Among them, glycolipids, particularly gangliosides (GGs) and sulfatides, are highly enriched in neuronal membranes and localized within lipid rafts, where they regulate receptor organization and signal transduction [19,20,21]. The disruption of glycolipid homeostasis is linked to both rare lysosomal storage disorders and common neurodegenerative diseases [22,23], influencing the aggregation and toxicity of proteins such as amyloid-β, tau, α-synuclein, and huntingtin [19].

Historically, research on glycolipids was limited by analytical challenges due to their chemical diversity and low abundance. Early techniques like thin-layer chromatography (TLC) and immunodetection lacked sensitivity and specificity [24,25].

**Figure 2:**
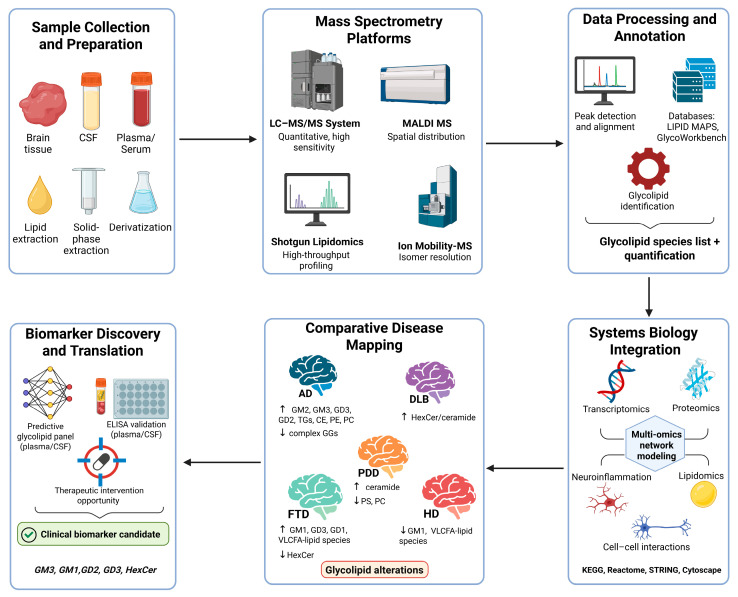
A systems workflow illustrating the integration of MS-based glycolipid profiling with bioinformatic and systems biology approaches to characterize glycolipid dysregulation in dementia (Created in BioRender. Sarbu, M. (2025) https://BioRender.com/n5tserg, accessed on 3 November 2025).

In this context, the development of modern mass spectrometry (MS), including liquid chromatography coupled with MS (LC-MS), matrix-assisted laser desorption/ionization MS imaging (MALDI-MSI), and ion mobility spectrometry (IMS), has revolutionized the field of lipidomics, enabling the detailed profiling of glycolipids across brain tissue, cerebrospinal fluid (CSF), and plasma. Hence, for a better overview, Table 2 presents the strengths and limitations of different MS platforms for glycolipid biomarkers. These technologies have also facilitated the integration of lipidomics with systems biology approaches, enabling network-based models that place glycolipid dysregulation within the broader molecular landscape of dementia (Figure 2).

MS-based studies consistently report reductions in sulfatides and alterations in GG composition in AD [34], disruptions in glycosphingolipid (GSL) metabolism in FTD [35], shifts in specific glycolipid species in DLB [36], and impaired GG biosynthesis in HD [37]. Importantly, these findings are detectable not only in the postmortem brain but also in biofluids, supporting their potential as minimally invasive biomarkers for early diagnosis, subtype differentiation, and therapeutic monitoring. Furthermore, glycolipids are increasingly viewed as active drivers of neurodegenerative cascades and promising therapeutic targets, with ongoing efforts focused on modulating their metabolism and interactions [38,39].

Integrating MS-based lipidomics with systems biology enhances diagnostic capability, enabling the discovery of disease-specific glycolipid signatures, predicting disease progression, and informing targeted therapeutic interventions.

The aim of this review is to provide an integrative overview of current evidence on GSL dysregulation in major forms of dementia, with particular emphasis on the findings generated by MS and systems biology approaches. We first summarize the fundamental roles of GSLs in neuronal physiology, followed by an examination of their profiles in AD and LBD (Lewy body dementia), including Parkinson’s disease (PD), FTD, HD, and mixed dementia. Methodological advances in lipidomics and systems-level analyses are then discussed, highlighting how these have expanded our understanding of lipid-mediated mechanisms in neurodegeneration. Finally, we outline emerging links between GSL metabolism, protein aggregation, neuroinflammation, and synaptic dysfunction and consider their implications for biomarker discovery and therapeutic strategies.

To conduct this review, a comprehensive literature search across PubMed, Google Scholar, and Web of Science yielded 412 articles (PubMed: 96; Google Scholar: 157; Web of Science: 159). After eliminating duplicates and excluding studies outside the defined scope, 171 articles remained for full review, with 53.80% published post-2020. By placing glycolipid research within the broader landscape of dementia biology and by emphasizing the methodological advancements that have enabled its recent progress, overall, this review highlights the major importance of GSLs in the pathogenesis of neurodegenerative disease and their potential as targets for biomarker discovery and therapeutic innovation.

## 2. Alzheimer’s Disease (AD)

AD is the most common form of dementia, accounting for 60–70% of cases [26,38,40,41]. AD predominantly affects women [42] and typically develops after age 65 [26]. Its prevalence has risen from 4.08 million cases in 1992 to 9.84 million in 2021 [43]. Characterized by cognitive decline, AD impacts individuals’ daily functioning and independence, through symptoms like memory decline, impaired thinking, and change in behavior [44,45].

AD prevalence increases dramatically with age; family history; genetic mutations; and environmental, metabolic, energetic, and vascular factors [46]. Although a conclusive diagnosis requires postmortem histopathology, current clinical diagnosis relies on neuropsychological testing and neuroimaging. Macroscopically, the neuronal and synaptic loss in AD is marked by cerebral atrophy [26,47,48], while microscopically, it is defined by abnormal protein aggregation, such as Aβ-peptide forming senile plaques (SPs) and phosphorylated tau (p-tau) protein creating neurofibrillary tangles (NFTs) and neuropil threads [49,50,51,52].

Aβ derives from the sequential β- and γ-secretase cleavage of amyloid precursor protein (APP) [38,51], forming insoluble fibrillar deposits linked to gliosis, neuroinflammation, and oxidative stress [53,54]. NFT burden correlates strongly with disease severity [55].

Studies suggest that the clinical symptoms of AD appear long after neurodegeneration, which involves many molecular changes [47]. As cell membrane breakdown occurs [47], lipid metabolism become dysregulated, affecting lipid raft structure and function [19,56]. Changes occur across lipid classes, including glycerolipids, glycerophospholipids, sphingolipids, and cholesterol [47,56]. GGs are also altered: reduced levels of GM1, GD1a, GD1b, and GT1b GGs, alongside increased levels of simpler species such as GM2, GM3, and GD3 and cholinergic markers, like Chol-1α (GQ1bα) and GT1aα, are associated with AD [19,48,51,56,57,58,59,60]. Monoclonal antibody A2B5 selectively stain neurons undergoing neurofibrillary degeneration and neuritic processes within SP in AD, recognizing c-series GG, such as GQ1c, that reappear abnormally in AD brains [61].

Technologies based on MS, particularly MALDI-MSI and TOF-SIMS [51,59], have become popular for lipid analysis in AD. Studies in APP/PS1 transgenic mouse models (TMMs) have consistently demonstrated significant reductions in sulfatides (SHexCers) and glycerophosphoinositols (GroPIns) in the cerebral cortex, hippocampus, and cerebellum, indicating both aging- and AD-related neurodegeneration [59]. A multimodal MS approach in a TMM (tgAPPArcSwe) [51] revealed a distinct lipid signature within amyloid plaques, characterized by a global depletion of cortical sulfatides and accumulation of plaque-specific lipids, including ceramide (d18:1/18:0), GM2, and GM3, containing 18:0 fatty acid moieties, in plaque peripheries and a relative enrichment inGM1 in plaque cores. These findings underscore the central role of glycolipid dysregulation in plaque microenvironments and highlight MSI as a powerful tool in identifying lipid-based biomarkers and therapeutic targets in AD.

LC-MS/MS studies have extended these insights to human tissues. Mechref et al. [62] reported dysregulated lipids in AD brains, particularly the upregulation of phosphatidylcholine (PC), phosphatidylglycerol (PG), ganglioside GD2, phosphatidylinositol (PI), phosphatidylserine (PS), lysophosphatidic acid, lysophosphatidylcholine, and sphingomyelin (SM), along with the downregulation of GD1a in AD.Such findings implicate disrupted phospholipid and sphingolipid pathways in AD pathophysiology and suggest glycosylation changes linked to disease progression [62,63,64]. Furthermore, investigations of human brain tissues uncovered alterations in glycan modifications and coregulated glycoform networks in AD, suggesting that specific glycosylation changes are closely linked to disease progression [62]. Together, these findings [62,63,64] demonstrate that integrating lipidomics and glycomics through LC-MS/MS profiling can reveal critical molecular signatures for early detection and potential therapeutic targeting in dementia.

Quantitative ultra-high-performance LC (UHPLC)-MS/MS lipidomics in *APP/PS1* mice identified 43 altered hippocampal lipid species, mainly glycerolipids, glycerophospholipids, and sphingolipids [56]. Elevated cholesteryl esters (CE 22:6, 22:4) promote Aβ aggregation and vascular dysfunction, while increases in PCs and phosphatidylethanolamines (PEs) indicate membrane remodeling. Declines in galactolipids and sphingolipids (hexosylceramide, HexCer, and ceramide, Cer) may impair membrane integrity and microglial function. The LC-MS profiling of GGs revealed 48 hippocampal species associated with AD, including potential biomarkers such as di-*O*-Ac-GT1a (d36:1) and *O*-Ac-GD1b (d36:1) [65]. MALDI-MSI studies further showed GM2 and GM3 accumulation near plaques correlating with *HEXA* gene expression, implicating impaired GG degradation [66].

Longitudinal MALDI-MSI in APP21 rats [67] and 5xFAD [68] mice highlighted age- and region-dependent shifts: simple GGs were elevated, complex GGs decreased, and the d20:1/d18:1 ratio increased due to the loss of d18:1 species. GM1 levels increased with age but decreased later in APP21 rats, while GM2 and GM3 accumulated, particularly GM3 (d18:1). High-resolution MSI demonstrated long-chain base-specific GG deposition within plaques: 18:1 species enriched subgranular zones; 20:1 localized to entorhinal pathways; and GM3 (d18:1/18:0), GM2 (d18:1/18:0), and GM1 (d18:1/18:0) colocalized with Aβ peptides [68].

Other MS studies demonstratedthe near-complete loss of ~20 GG species in the AD cerebellum and reductions in the right cerebral hemisphere, paralleling Aβ deposition and neuronal loss [69,70]. Human MSI confirmed region-specific GM1 alterations, notably reduced GM1 (d20:1)/(d18:1) ratios [45,58]. TLC/MALDI-TOF-MS corroborated decreases in GD1b and GT1b with the predominance of d18:1-containing GGs [71]. Serum LC-MS revealed CE (16:3) and GM3 (d18:1/18:1) as promising biomarkers for AD severity [72].

Autoantibodies against GGs also contribute to pathology. Elevated IgM and IgG antibodies against GM1, GD1b, GT1b, GQ1b, and cholinergic-specific GQ1bα were found in AD sera and in the cortex and hippocampus, potentially impairing cholinergic signaling and serving as early immunological biomarkers [42].

**Figure 3:**
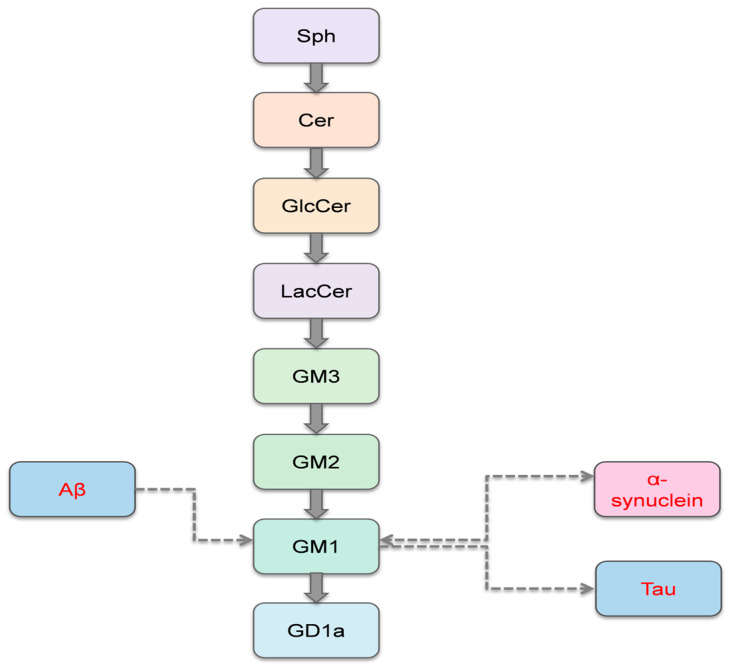
Metabolism of glycosphingolipids and their interactions with proteins.

Several studies demonstrated that GM1 critically modulates AD pathogenesis by promoting Aβ aggregation and senile plaque formation. Elevated GM1 induces conformational alterations in γ-secretase, enhances Aβ production, and stabilizes GAβ complexes that insert into the lipid membrane [44,54]. Cholesterol-rich lipid rafts potentiate GM1-Aβ interactions via hydrogen bonding with GM1′s glycosidic linkage, accelerating peptide binding [73]. Structural studies further revealed that Aβ and α-synuclein recognize specific GGs via a shared motif; engineered peptides mimicking this motif can bind GM1 and block Aβ neuronal uptake, suggesting therapeutic potential [74]. A diagram illustrating the metabolism of GSLs and their interactions with key proteins, Aβ, α-synuclein, and tau, is presented in Figure 3.

Finally, detecting glycolipid-associated protein complexes, such as GM1-bound Aβ [75], apolipoprotein E isoforms [76], clusterin [77], complement C1q [78], and low-density lipoprotein receptor-related protein 1 [79], via immunoassays (ELISA, electrochemiluminescence, Luminex) bridges MS discoveries with clinical application [80]. GGs remain among the most valuable lipid biomarkers in AD, with region-dependent reductions: in early-onset AD, declines are prominent in gray and frontal white matter, whereas sporadic AD shows reductions mainly in the temporal cortex, hippocampus, and frontal regions [59].

## 3. Lewy Body Dementia (LBD)

LBD represents an umbrella term that includes both dementia with Lewy bodies (DLB) and Parkinson’s disease dementia (PDD). LBD is recognized as the second most widespread form of degenerative dementia in older people after AD [81].

### 3.1. Dementia with Lewy Bodies (DLB)

DLB accounts for approximately 20% of cases [82] and is pathologically defined by the misfolding and aggregation of α-synuclein into Lewy bodies and neurites, leading to the disruption of synaptic integrity, neurotransmission failure, and progressive neuronal loss across cortical and subcortical regions [83]. Normally involved in synaptic vesicle cycling, α-synuclein aggregation disrupts network connectivity and cognition. Currently, DLB diagnosis is primarily clinical, relying on fluctuating cognition, REM sleep behavior disorder, parkinsonism, and visual hallucinations. While imaging modalities such as fluoro-deoxyglucose positron emission tomography (FDG-PET), revealing occipital hypometabolism or the cingulate island sign, and dopamine transporter–single-photon emission computed tomography (DAT-SPECT), showing reduced striatal dopamine transporter uptake, support diagnosis, biomarker interpretation is challenged by frequent copathology with AD, especially Aβ and tau deposition [84,85]. Despite these advances, definitive diagnosis remains postmortem, underscoring the need for molecular biomarkers capable of differentiating DLB from AD and PDD during one’s lifetime, which is essential for targeted management.

Initial biomarker studies targeting α-synuclein in CSF and plasma produced inconsistent results due to methodological variability [86,87,88]. Real-time quaking-induced conversion assays represent a major advancement, enabling the amplification and detection of misfolded α-synuclein aggregates with high diagnostic accuracy, including the clear differentiation of DLB from AD [89,90,91]. Additional candidates, including the Aβ42/40 ratio, p-tau, neurofilament light chain (NfL), and neurotransmitter metabolites, show supportive rather than definitive diagnostic value [92,93,94,95,96].

Machine learning (ML) integrated with MS-based lipidomics offers new diagnostic potential. Hence, by using UPLC-MS combined with ML, Shen et al. [36] identified distinct plasmalipidomic signatures discriminating DLB from healthy controls and AD, particularly alterations in sphingolipid (sphingoid bases, ceramides, and monohexosylceramides (Hex1Cers)) metabolism. A 13-lipid panel, including Hex1Cer (d18:1/24:0) and Hex1Cer (d18:1/23:0), showed high predictive accuracy. Across analyses, ceramides, sphingosines, PE, and PC emerged as the most consistently dysregulated lipid classes in DLB. These findings suggest that disruptions in sphingolipid signaling and membrane phospholipid remodeling may contribute to the pathophysiology of DLB [36].

Earlier plasma lipidomic studies also reported elevated plasma ceramides (16:0, 18:1, 20:0, 24:1) and Hex1Cer (18:1, 24:1) in both AD and DLB compared with controls, though they lacked disease specificity, indicating that systemic sphingolipid changes may reflect general neurodegenerative processes [97]. In contrast, CSF analyses revealed more distinct biochemical patterns. Lerche et al. [98] reported reduced galactosylsphingosine (GalSph) and ceramide levels in DLB compared with PD and controls, independent of *GBA1* variant status, indicating more specific lipid alterations in DLB pathology.

Although the full potential of MS-based GSL profiling in DLB remains underexplored, current evidence highlights its value in uncovering subtle disease-specific lipid alterations and its promise as a non-invasive biomarker strategy. Plasma lipidomics reflects broader neurodegenerative changes, whereas CSF sphingolipid profiling appears to capture more specific biochemical signatures of DLB. The integration of MS-based lipidomics with ML approaches thus represents a powerful framework for enhancing differential diagnosis and deepening insight into the molecular mechanisms underlying DLB.

### 3.2. Parkinson’s Disease Dementia (PDD)

PD is the second most common neurodegenerative disorder after AD, pathologically characterized by dopaminergic neuron loss in the substantia nigra and the aggregation of misfolded α-synuclein into Lewy bodies, placing it among the α-synucleinopathies [99,100]. Because clinical diagnosis typically occurs after substantial neuronal loss, there is an urgent need for biomarkers that enable earlier and more accurate detection [101]. Cognitive decline represents a major non-motor complication of PD, while PDD affects most patients in later stages [102,103]. Although PDD and DLB share overlapping α-synuclein pathology, they differ mainly in the temporal sequence of cognitive and motor symptoms [104]. The onset of dementia in PD signals accelerated disease progression and increased mortality [105].

Following these clinical needs, high-throughput proteomics has provided important molecular insights into PD and PDD pathology. MS-based studies have revealed the post-translational modifications (PTMs) of key neurodegenerative proteins such as tau, α-synuclein, Aβ, and TDP-43 across various diseases [106,107,108,109].

In biofluids, phosphorylated and truncated tau and modified Aβ species can also be detected [106,107,110], demonstrating the ability of MS to resolve disease-specific proteoform diversity. However, most PTM-based biomarker research has concentrated mainly on AD and DLB, leaving PDD relatively underexplored. This gap has prompted growing interest in lipidomics and glycolipidomics as complementary molecular domains that may better capture the metabolic aspects of cognitive decline.

Building on this proteomic foundation, recent research has increasingly focused on lipidomic signatures, particularly GSL metabolism, as a promising field for PDD biomarker discovery. Targeted LC-MS/MS studies have revealed elevated plasma ceramide and glucosylceramide (GlcCer) levels in sporadic PD, correlating with poorer cognitive performance [111,112]. Using a validated HPLC-MS/MS platform, Xing et al. [112] identified specific ceramide species, namely 24:1 and 14:0, negatively associated with verbal memory, while 22:0 and 20:0 correlated with hallucinations and anxiety. These relationships remained significant after adjusting for confounders, suggesting that distinct ceramide glycoforms may serve as biomarkers of domain-specific cognitive and neuropsychiatric dysfunction in PDD.

Extending beyond these targeted analyses, high-resolution lipidomics has provided additional insights into PD progression and dementia risk. Zardini Buzatto et al. [105] applied a high-sensitivity LC-QTOF-MS lipidomics workflow to evaluate whether baseline serum lipid profiles could predict cognitive decline in PD. Over a three-year follow-up, they found that patients who later developed dementia exhibited elevated ceramides, diacylglycerols, and triacylglycerols, together with reduced PCs, bis(monoacyl)glycerophosphates (BMPs), and PSs, distinguishing them from patients who remained cognitively stable. Notably, multivariate models (PLS-DA, OPLS-DA, Random Forest) achieved excellent discrimination, with a five-lipid biomarker panel yielding an area under the curve of 0.993 with >95% accuracy in validation, demonstrating that serum lipidomic signatures can robustly predict dementia years before clinical onset.

Integrating MS-based sphingolipidomics with artificial intelligence (AI) and ML further enhances predictive precision. ML models combining LC-MS lipidomics with clinical data can forecast motor and non-motor trajectories up to two years in advance and identify lipid signatures associated with disease severity [113,114]. In summary, MS-based sphingolipidomics and AI-driven analyses provide compelling evidence that specific ceramide species and broader lipidomic signatures are tightly associated with cognitive and neuropsychiatric manifestations in PDD [105,112].

Overall, GSL metabolism represents a promising biomarker domain that complements protein-based markers such as α-synuclein and NfL, underscoring the translational potential of MS lipidomics for early stratification and therapeutic monitoring. Ultimately, continued research integrating lipidomics, proteomics, and advanced computational tools will deepen the understanding of PD and its progression to dementia. Future multi-omics studies correlated with clinical and imaging outcomes are essential to validate lipid-based biomarkers and develop practical diagnostic and prognostic tools, thereby supporting precision medicine in PD.

## 4. Frontotemporal Dementia (FTD)

FTD, historically referred to as Pick’s disease, comprises a heterogeneous group of non-Alzheimer’s neurodegenerative dementias, primarily affecting the frontal and temporal lobes, leading to progressive impairment in behavior, language, and executive function [115,116]. FTD represents the third most common neurodegenerative dementia after AD and DLB and the second leading cause of dementia in individuals under 65 years [117].

Clinical overlap with psychiatric disorders complicates diagnosis, underscoring the need for reliable fluid biomarkers to enable early detection and disease stratification. Genetic mutations, most commonly in *GRN*, *C9orf72*, and *MAPT*, account for 15–20% of FTD cases and highlight the role of lysosomal dysfunction and impaired lipid homeostasis in disease pathogenesis [118].

Beyond conventional biomarkers, such as NfL and progranulin (PGRN), glycolipid and sphingolipid profiling via MS provide mechanistic insight into membrane biology and neuronal–glial interactions and lipid-mediated signaling.The ability to detect these lipid alterations in peripheral biofluids through MS expands the potential for minimally invasive biomarkers and precision medicine approaches in FTD and related syndromes [22,119,120].

Recent lipidomic studies reveal both the shared and mutation-specific mechanisms of lipid dysregulation in FTD. In 2022, Boland et al. [121] demonstrated that *GRN* haploinsufficiency disrupts lysosomal homeostasis by reducing BMP levels, leading to GG (GM1, GD3, and GD1) accumulation in *GRN*-associated FTD-TDP. GT1 reduction was specific to *GRN*-FTD, and BMP supplementation normalized GM2 levels in *PGRN*-knockout cells, linking lysosomal dysfunction to lipid accumulation [121]. Similarly, increased GT1a and/or GD2, in Pick’s disease brains, indicates impaired GSL clearance in FTD [122].

Expanding this theme, Kim et al. [123] identified altered plasma lipid signatures in behavioral variant FTD (bvFTD), characterized by elevated triacylglycerol (TG) and reduced PS and PG, along with specific individual lipid species, such as TG (16:0/16:0/16:0), diglyceride DG (18:1/22:0), PC (32:0), PS (41:5), and SM (36:4), capable of distinguishing bvFTD from AD and healthy controls [123].

Given earlier evidence that *GRN* mutations interrupt lysosomal lipid catabolism, Marian et al. [124] compared lipid metabolism across *GRN*-FTD and *C9orf72-*FTD postmortem brains, revealing pronounced myelin lipid loss, cholesterol ester accumulation, and fatty acid metabolism disruption that was more pronounced in *GRN*-FTD, consistent with its more severe white matter pathology. Extending these tissue-based findings to a peripheral biomarker context, Marian et al. [120] found reduced plasma myelin-enriched glycolipids (HexCer), especially 22:0 GlcCer and GalCer that correlated inversely with disease duration and white matter damage suggesting their utility as peripheral biomarkers of FTD severity.

Mechanistically, PGRN deficiency also reduces β-glucocerebrosidase (GCase) activity and impairs enzyme maturation in FTD-*GRN* brains [125], linking enzyme-level lysosomal dysfunction with sphingolipid imbalance. Together, GG accumulation [121], myelin lipid loss [124], and plasma HexCer reductions [120] outline a coherent cascade in which *GRN*-related lysosomal defects drive both central and peripheral lipid abnormalities. Adding further complexity, He et al. [126] identified significant elevations of very-long-chain fatty acid (VLCFA)-containing phospholipids, particularly PC (30:5/18:1), PE (33:4/20:4), and PE (33:4/22:6), in the FTD cortex, correlated with *ELOVL4* expression, implicating aberrant phospholipid elongation in disease mechanisms.

Taken together, these convergent findings delineate that lipid dysregulation in FTD is multifaceted, spanning GG accumulation, sphingolipid and myelin lipid loss, lysosomal enzyme dysfunction, and VLCFA-containing phospholipid elevations. Importantly, both central (brain tissue) and peripheral (plasma) lipid changes show potential as diagnostic and prognostic biomarkers while offering mechanistic insights into disease pathogenesis and therapeutic targets.

## 5. Huntington’s Disease (HD)

HD is a monogenic, autosomal dominant neurodegenerative disorder caused by the expansion of a Cytosine–Adenine–Guanine trinucleotide repeat in the *HTT* gene on chromosome 4, leading to an expanded polyglutamine tract in the HTT protein. Clinically, HD presents with motor, cognitive, and psychiatric symptoms that typically emerge in mid-adulthood after a prodromal phase [127]. Because the causal mutation is known, HD provides a unique model for investigating presymptomatic and early symptomatic stages, enabling the identification of biomarkers reflecting early molecular alterations and therapeutic response [128].

Cognitive impairment is an integral component of HD and progresses toward Huntington’s disease dementia (HDD) in advanced stages [129]. Subcortical cognitive deficits, especially in attention, executive function, and psychomotor speed, correlate with functional decline and dementia progression [130,131], underscoring the need for biomarkers that reflect early molecular changes, disease severity, or response to therapies.

MS-based lipidomics has revealed widespread metabolic dysregulation in HD. The untargeted UHPLC-MS/MS of plasma and CSF identified altered ceramides, HexCers, SMs, diacylglycerols, and PCs, which correlated with cognitive scores from the Stroop and Verbal Fluency tests [132]. Complementary MALDI-MSI studies of HD brain tissue revealed widespread sphingolipid and phospholipid dysregulation in the caudate and cortex, indicating disrupted neuronal membrane integrity, a shift from very-long-chain to long-chain species, and structural changes with dementia-related network dysfunction [133,134,135].

Within the GSL family, GM1 has emerged as a key molecule linking lipid metabolism to synaptic and cognitive vulnerability in HD. Multiple MS-based and cellular studies have shown markedly reduced GM1 levels in HD patient-derived fibroblasts and neural cultures [136], while GM1 supplementation restored synaptic function and improved motor and cognitive outcomes in HD mouse models [137,138]. These findings implicate GM1 deficiency as both a mechanistic biomarker and a potential therapeutic target for HD-related cognitive decline.

Mechanistically, lipids such as ceramides and GSLs function as bioactive signaling molecules that regulate apoptosis, synaptic transmission, and neuroinflammatory responses. Their dysregulation in HD links systemic metabolic stress to synaptic and cognitive dysfunction. While protein biomarkers such as NfL and mutant huntingtin (mHTT) remain robust indicators of axonal injury and genetic burden [133,139], they do not capture the lysosomal and membrane lipid abnormalities revealed by MS. Integrating proteomic and lipidomic markers may therefore enhance diagnostic precision and prognostic power for cognitive decline. Consistent with these findings, MALDI-IMS and LC-MS analyses of postmortem HD brain tissue revealed sphingolipid chainlength alterations and regional phospholipid depletion that impair neuronal connectivity in executive and memory networks [134].

A major limitation of current HD lipidomic research is that most studies remain cross-sectional, limiting the assessment of predictive value for conversion from prodromal to dementiastages. By contrast, longitudinal MS-based lipidomics in PD has successfully identified predictive biomarker panels for incipient dementia [105]. Implementing similar longitudinal MS-based approaches in HD could enable the early stratification of gene-positive individuals and guide preventive interventions. Recent advances in AI and ML offer new opportunities to enhance HD biomarker discovery and clinical prediction. AI-driven models integrating multimodal MS-based data have shown promise for improving prognostic accuracy and patient stratification in early-stage HD [140,141]. MS-based approaches, including LC-MS/MS, UHPLC-MS, and MALDI-IMS, consistently reveal alterations in ceramides, SMs, and GSLs that connect membrane lipid metabolism with cognitive decline [133,134,136]. In particular, GM1 depletion emerges as both a mechanistic biomarker of synaptic vulnerability and a potential therapeutic target [136,137,138]. Consequently, future longitudinal multi-omics studies integrating AI and ML-driven analytics will be crucial to validate GSLs and other lipidomic candidates as predictive and clinically actionable biomarkers of HDD.

## 6. Mixed Dementia

Mixed dementia is a frequent and biologically heterogeneous form of cognitive impairment in which two or more neuropathological processes coexist often with synergistic effects on cognitive decline; most typically, these are AD proteinopathies [6]. Additional copathologies such as LB or TDP-43 inclusions are contributors that increase clinical complexity and disease progression [142].

Autopsy series and population studies indicate that mixed pathologies become prevalent with advancing age and that coexisting vascular and neurodegenerative lesions account for a substantial fraction of dementia cases in older adults. Consequently, a significant proportion of clinically diagnosed AD or vascular dementia (VD) actually represents mixed forms [6,143].

Clinically, mixed dementia often presents features of both component disorders: the episodic memory impairment characteristic of AD, as well asthe executive dysfunction, slowed processing speed, attention deficits, gait disturbance, and focal neurological signs associated with vascular contributions. When Lewy Body or TDP-43 pathology is also present, supplementary signs such as visual hallucinations, parkinsonism, or disproportionate temporal–hippocampal neuronal loss with hippocampal sclerosis occur, producing a phenotype that can shift over time as the different pathologies evolve [144].

Epidemiologically, mixed dementia incidence rises steeply with age and isassociated with vascular risk factors [145]; hence, creating strategies that target these factors at the population level isa major public health method for preventing this disease [146].

Modern diagnostic approaches attempt to deconvolute the contributions of degenerative and vascular processes in vivo by combining clinical assessment with (i) structural magnetic resonance imaging (MRI) to visualize infarcts, lacunes, white matter hyperintensities, microbleeds, and atrophy [147]; (ii) positron emission tomography (PET) for amyloid and tau [148]; (iii) CSF assays [149] of Aβ42 or Aβ42/40 or total and p-tau; and (iv) increasingly sensitive plasma biomarkers, such as p-tau217, p-tau181, Aβ ratios, and NfL, that provide scalable screening options [150].

These molecular tools increase specificity for AD and, in conjunction with MRI, are able to identify cases where vascular lesions and AD biomarkers coexist, although important limitations still remain. For instance, MRI underestimates minor cortical microinfarcts and diffuse small-vessel damage, PET is costly and not universally available, CSF sampling is invasive, plasma assays are influenced by peripheral factors and assay platform variability, and biomarker thresholds derived from pure disease cohorts may perform differently in mixed populations.

From a therapeutic perspective, treatment is focused on managing vascular risk factors [151,152] and the administration of AD drugs. While cholinesterase inhibitors and memantine may offer limited benefits [153], new anti-amyloid therapies are promising though remain unproven in mixed cohorts [154]. At the molecular level, mixed dementia arises from overlapping pathogenic cascades. In AD, abnormal APP processing generates toxic Aβ oligomers and plaques that induce oxidative stress and neuroinflammation, while hyperphosphorylated tau disrupts microtubules and neuronal integrity [155]. Vascular processes cause ischemia, blood–brain barrier (BBB) disruption, and inflammation, further amplifying neural injury.

Importantly, all these pathways interact: (i) vascular injury impairs the perivascular and endothelial clearance of Aβ, promoting its accumulation; (ii) Aβ deposition in vessels directly injures endothelial cells and pericytes; and (iii) apolipoprotein E ε4 modulates both amyloid accumulation and vascular integrity since ε4 carriers exhibit earlier BBB breakdown and pericyte dysfunction that can precede overt amyloid or tau pathology, creating an environment leading to mixed dementia.

Downstreamshared mechanisms across proteinopathies and ischemia include mitochondrial dysfunction, calcium dysregulation, chronic microglial and astrocytic activation, the failure of proteostatic systems, and metabolic shifts away from efficient oxidative phosphorylation toward impaired glycolysis, all of which converge to produce synaptic failure and neuronal death [156].

These molecular insights motivated an expanded biomarker agenda to include lipidomic and glycolipidomic signatures that reflect membrane, synaptic, and myelin integrity. Because GSLs participate in synapse formation and signal transduction, and modulate neuroinflammation and cell–cell interactions, their dysregulation reflects both degenerative and vascular/myelin damage and therefore holds promise as a biomarker set for mixed dementia [157].

In recent years, MS has been central to modern biomarker discovery and validation in neurodegenerative diseases because it enables the sensitive, high-resolution, and relatively unbiased profiling of biomolecules in brain tissue, CSF, and plasma without relying solely on antibody reagents [158,159,160,161]. Specific lipidomic studies of mixed dementia and related disorders illustrate these points. Hence, a comparative LC-MS lipidomic analysis of white and gray matter from the temporal cortex of subcortical ischemic vascular dementia patients, mixed dementia patients, and controls found pronounced alterations in sphingolipid classes and ceramides, as well as increases in some GG species, i.e., GM3 and markers of membrane breakdown, in mixed dementia white matter compared with controls. These features are consistent with the combined effects of neuronal degeneration and myelin/axonal injury in mixed pathology [162]. Such tissue-level findings provide mechanistic plausibility for glycolipid markers in biofluids and suggest candidate species for further study.

Additional MS-based investigations mapped GG distributions to Aβ plaques and periplaque regions and demonstrated the age- and disease-related dysregulation of GG degradation pathways with increases in GM2/GM3 in plaque-rich areas [66]. The results linked lipidomic profiles to imaging indices of vascular injury and white matter lesion burden, evidencing that lipid changes reflect the spatial interplay of degenerative and vascular lesions.

On the other hand, new developments in MALDI-MSI and related workflows, such as quantitative MSI combined with on-tissue extraction and LC-MS/MS, presently enablethenear-cellular mapping of lipids, permitting the direct visualization of how glycolipid perturbations colocalize with plaques, microinfarcts, or gliotic regions in human brain sections and model systems [163].

The importance of glycolipids as biomarkers derives from both biology and analytic feasibility: (i) GGs and GSLs directly report on membrane/synaptic composition and myelin health; (ii) they are sensitive to enzymatic shifts in lipid metabolism that occur with aging, hypoxia, and inflammation; and (iii) some species can be measured in CSF and, with advanced techniques, even in plasma. In AD specifically, Noel et al. [164] discovered altered GG patterns, including changes in GM1, GM2, GM3, GD1a/b, and GT1b and shifts in sulfatides and ceramides that correlate with amyloid and tau pathology or cognitive decline. In vascular and mixed dementias, white matter lipid degradation products and altered sphingolipid ratios reflect ischemic/myelin injury and overlap with degenerative signatures in mixed cases, suggesting that panels of glycolipids together with protein biomarkers could enhance sensitivity and specificity for mixed pathology [165].

In the past few years, modern and high-performance MS technologies have made several concrete achievements in glycolipid biomarker discovery for mixed dementia: (i) methodological refinements in extraction and chromatographic separation now permit the reliable detection and partial isomer resolution of GG species differing by sialylation state, ceramide backbone length, and unsaturation; (ii) high-resolution TOF instruments combined with MS/MS fragmentation and IMS allow for the structural assignment of glycan and lipid moieties at unparalleled sensitivity and reproducibility and with a wealth of compositional and structural data; (iii) MALDI-MSI and DESI imaging approaches enable spatially resolved lipidomics, linking molecular changes to histopathology in the same tissue section; and (iv) targeted LC-MS/MS assays developed for CSF and plasma enable quantitative panels suitable for larger cohort studies and longitudinal sampling, altogether moving glycolipid candidates from tissue discovery toward biofluid validation. Nevertheless, translation faces challenges such as inter-laboratory standardization; the low abundance and peripheral dilution of brain-derived lipids in plasma; confounding by diet/peripheral metabolism and renal function; and the need for large, well-phenotyped mixed dementia cohorts with paired imaging and neuropathology for validation [17].

Below, recent glycolipid biomarker candidates compiled in the concise Table 3 from MS-based tissue and biofluid investigation of species that have been implicated across studies in AD, VD, and mixed dementia are presented. Table 3 lists the representative glycolipid classes and specific molecular species that have appeared repeatedly as altered in disease cohorts; the table does not represent an exhaustive inventory but rather a prioritized set based on reproducible reports and biological plausibility in mixed pathology.

In glycolipidomics of mixed dementia, the emerging guidance from methodological investigations and discovery studies supports the following practical strategies: (i) the use of untargeted high-resolution IMS, LC-MS, or MSI with IMS for initial discovery in well-phenotyped brain tissue, paired with histology and proteomics to connect lipid changes to plaques, gliosis, and microinfarcts; (ii) the prioritization of candidate species that are abundant enough and biochemically plausible for measurement in CSF and the development of targeted LC-MS/MS selected reaction monitoring assays with isotopically labeled internal standards for validation in CSF/plasma cohorts; (iii) performing, whenever possible, matched imaging using MRI and fluid biomarker inventory, i.e., Aβ/tau/NfL plus a glycolipid panel, in order to determine whether glycolipid signals add diagnostic or prognostic value in mixed dementia beyond classical markers; and (iv) the standardization of sample collection, extraction, and instrument methods across centers to enable multi-site validation and regulatory qualification.

In conclusion, the advancements of MS and allied systems biology techniques such as high-resolution LC-MS/MS, IMS-MS, MALDI-MSI, and hybrid quantitative imaging workflows allow for the discovery of glycolipid biomarkers and targeted measurement.

The pathway forward requires large longitudinal cohorts with detailed clinical, imaging, and neuropathological characterization; standardized MS protocols; and multimodal MS which pairs MSI, high-resolution IMS, or LC-MS [160] and multi-omics integration to improve the diagnosis, prognostics, and monitoring of mixed dementia and enable trials of combinatorial therapies.

## 7. Cross-Dementia Comparison of GSL Profiles: Insights and Limitations

GSLs have emerged as critical modulators of neurodegenerative processes across multiple dementia subtypes, reflecting both the conserved and disease-specific mechanisms of pathology.

In AD, high-resolution MS studies consistently show reductions in GM1, GD1a, GD1b, and GT1b across key regions such as the hippocampus and frontal and temporal cortices. These losses are often accompanied by the accumulation of simpler structures, particularly GM2, GM3, and GD3, localized to the peripheries of Aβ plaques. These alterations facilitate the aggregation of both Aβ and tau, highlighting the role of GGs in both initiating and propagating AD pathology. Moreover, subtle changes in the ratio of long-chain base species, such as d18:1 versus d20:1, influence Aβ binding dynamics and plaque composition, suggesting that minor lipidomic variations can critically shape disease progression.

Despite the insights provided by these studies, several methodological and interpretative challenges exist. Many MS-based analyses rely on postmortem tissue, limiting the ability to assess longitudinal changes and early-stage disease processes. Animal models often exaggerate plaque pathology relative to humans, potentially inflating observed GSL alterations. Inter-study variability is common, reflecting differences in GSL extraction and purification methods, MS performances and parameters, and data normalization. Systems biology approaches suggest that GG loss in AD is coupled to the dysregulation of synaptic proteins, APP-processing complexes, and neuroinflammatory mediators. However, establishing causality between GG alterations and neurodegeneration remains a challenge, as lipid changes may represent both pathogenic drivers and compensatory responses.

FTD exhibits a distinct GSL signature relative to AD. GM1, GD1a, and GT1b are reduced in frontal and temporal white matter, whereas hippocampal regions are relatively spared. Notably, c-series GGs, including GQ1c and GT3, may accumulate in degenerating neurons, potentially reflecting compensatory mechanisms responding to tau-mediated cytoskeletal destabilization. Unlike AD, the accumulation of simple GGs such as GM2 and GM3 is less prominent, implying that the deposition of simple GGs is less central to FTD pathophysiology. This aspect highlights a critical distinction between tau-dominant dementias and amyloid-centered disorders: in FTD, GGs are linked to intracellular cytoskeletal disruptions rather than extracellular protein aggregation. Nevertheless, most insights derive from animal models or small patient cohorts; hence, regional heterogeneity may obscure broader systemic patterns.

DLB and PDD illustrate how disrupted GSL metabolism contributes to α-synuclein pathology. Simple GGs, particularly GM2 and GM3, promote Lewy body formation, while the depletion of GM1 and GD1a impairs lipid raft integrity, worsening cognitive and motor deficits. MSI shows region-specific GSL changes linked to symptom profiles, though distinguishing disease effects from aging remains challenging. Overall, alteredendocytosis, vesicular trafficking, and neuroinflammation appear to be related to the GSL-α-synuclein connection

HD shows early and severe deficits in GGs such as GM1, GM2, GD1a, and GD1b likely driven by mHTT-related disruptions in vesicular trafficking and signaling. Importantly, these changes may precede neurodegeneration and serve as early biomarkers, though most evidence comes from animal models. The causal link between GG loss and mHTT aggregation remains unclear, and human validation is limited. Furthermore, integrated analyses suggest that GG disturbances contribute to cytoskeletal, mitochondrial, and synaptic dysfunction underlying cognitive and motor decline.

Mixed dementia, a combination of AD and vascular pathology, exhibits synergistic GSL dysregulation. The early accumulation of GM3 and GM2 is observed in ischemic or hypoperfused regions, while the progressive depletion of GM1, GD1a, and GT1b mirrors amyloid-driven cortical and hippocampal degeneration. However, deciphering the contributions of vascular injury vs. amyloid pathology remains challenging, particularly due to the lack of standardized postmortem tissue protocols and longitudinal imaging approaches.

A comparative perspective across dementias highlights that complex GG reduction, especially GM1, GD1a, GD1b, and GT1b, is a common feature, likely contributing to lipid raft destabilization and synaptic vulnerability. Conversely, the accumulation of simple GGs such as GM2 and GM3 is prominent in AD, DLB, and mixed dementia but less evident in FTD, reflecting disease-specific proteinopathies and regional susceptibilities. C-series GGs, including GQ1c, appear selectively in FTD and AD, potentially marking tau-associated degeneration. Furthermore, shifts in long-chain base composition (d18:1 vs. d20:1) in AD affect plaque-associated GG distributions, revealing subtle lipidomic remodeling as a determinant of protein aggregation kinetics. These observations demonstrate the value of disease-specific glycolipidomic profiling for uncovering the mechanisms of neurodegeneration.

Overall, from amethodological point of view, MS has revolutionized the study of GSLs in dementia, providing unparalleled sensitivity, specificity, and resolution. MALDI-MSI, electrospray ionization (ESI)–MS and LC-MS/MS enable the detection of both quantitative and spatial changes in their composition, allowing for correlations with specific brain regions. However, limitations persist in all studies, and they are related to postmortem tissue degradation, inter-study variability, and the challenge of integrating MS data across platforms. Also, the cross-sectional nature of many studies limits insights into temporal dynamics, while animal models may not fully reproduce human disease, particularly for the cases of dementia. Moreover, systems biology is able to integrate glycolipid alterations with molecular networks to reveal potential therapeutic hubs, though translating these insights into clinical strategies remains challenging. Therapeutically, GSLs represent promising targets. In AD, GM1 supplementation modulated Aβ aggregation and enhanced neurotrophic signaling. Preclinical studies in HD and PD models suggest similar benefits, with GM1 restoring synaptic function and mitigating protein aggregation. Beyond direct supplementation, the modulation of GG metabolism via glycosyltransferase inhibitors, sphingolipid-targeted enzymes, or small molecules that stabilize specific GG species offers potential alternatives for disease-modifying interventions.

Finally, all findings indicate that, overall, GSLs act as both shared and disease-specific drivers of dementia, where common complex GG depletion contributes to synaptic vulnerability; however, GG patterns vary across diseases.

## 8. Conclusions and Perspectives

Research into the role of GSLs in dementia is beginning to redesign our perspectives on the molecular bases of neurodegenerative diseases. Data collected from multiple studies now clearly indicate that GSLs are not just inactive compositional building blocks of neuronal membranes but dynamic regulators of brain processes. As discussed above, the recurring patterns indicate core weaknesses in neuronal GSL regulation, whereas the distinct changes cause the diverse symptoms and progression seen across different dementias. In this context, Table 4 presents a synthetic view on the altered GSL expression specific to each dementia type discussed, along with possible biomarkers.

Technological refinements in MS have been crucial in enabling recent discoveries in glycolipid research, providing acritical basis for discovering novel lipid signatures in dementia, and reshaping our understanding of glycolipid biology. By offering unprecedented sensitivity, resolving power, and detailed structural information, modern MS has created opportunities to map complex lipid alterations with high accuracy and depth.

Up-to-date MS-based analytical platforms have the capability to detect and characterize a wide range of glycolipids with far greater precision than before, allowing us to build detailed maps of lipid changes in brains affected by dementia. When these lipidomic datasets are connected with systems biology approaches, they can be placed into larger networks of the processes involving other biomolecules. This kind of integrative view is crucial since dementia is not driven by a single factor but emerges from the intersection of many disrupted pathways.

Several promising pathways emerge when considering future directions. Hence, one key priority is to establish whether glycolipid changes occur early enough to serve as warning signals before clinical symptoms appear. This would provide the opportunity to use these glycoconjugates as biomarkers for risk assessment and early diagnosis. Equally critical is the alignment of methodologies and protocols across the laboratories involved in the study of dementia-associated glycolipids in order to generate results that are reliably compared, reproducible, and validated in larger patient cohorts.

Beyond description, there is also a pressing need to move toward mechanistic studies that clarify how specific glycolipid changes influence neuronal survival, immune responses, and protein aggregation.

Noteworthy also are the potential therapeutic implications and prospects. Hence, if certain glycolipids prove to be drivers rather than bystanders in dementia, they could become targets for interventions aimed at stabilizing neuronal membranes, modulating inflammation, or preventing toxic protein buildup. Even if they are not direct drivers, their measurable changes could complement other diagnostic tools, contributing to more precise personalized treatment strategies.

Moreover, the progress of AI presents opportunities for glycolipid research in dementia, facilitating the integration and analysis of complex datasets. ML algorithms can uncover subtle patterns in high-dimensional lipidomic data that might elude conventional analysis, linking glycolipid changes to specific disease stages or phenotypes. AI-driven models can integrate lipidomic profiles with other omics layers, such as genomics, proteomics, and transcriptomics, to construct systems-level maps of dementia pathophysiology.

When combined with MS, the potential of AI expands even further. MS produces vast, highly detailed datasets, while AI can process them at scale, revealing patterns and correlations that remain hidden to traditional approaches. This synergy enables a more precise mapping of glycolipid alterations across dementia types, enhancing biomarker discovery and deepening mechanistic insight. AI is also able to greatly improve MS workflows by automating data annotation, the interpretation of spectra, and structural identification while predicting the functional consequences of the detected changes in the glycolipid expression pattern.

In conclusion, MS and systems biology have developed to the point where detailed molecular insights can be translated into practical clinical advances. Based on ongoing research, which aims to connect novel findings at the molecular level with clinical outcomes, this research direction has the potential to significantly improve the methods of early dementia detection and its treatment.

## Figures and Tables

**Table 1:** Key features of different dementia types [4,5,6,7,8,9,10,11,12,13,14,15,16].

	Disorder	Dementia of Alzheimer’s Type (AD)	Dementia with Lewy Body (DLB)	Frontotemporal Dementia (FTD)	Parkinson’s Disease Dementia (PDD)	Huntington’s Disease (HD)	Mixed Dementia
Features							
Onset		Presenile or senile	Senile	Presenile	Late onset	Presenile	Senile onset
Age at diagnosis		<65 s or >65 s	50 s–80 s	40 s and early 60 s	>70 s	30 s or 40 s	>65
Patient profile		Predominantly female	Slight male predominance	Male predominance	Male predominance	No gender preference	No gender preference
Brain abnormalities		Accumulation of amyloid plaques and tau tangles	α-synuclein aggregation in cortical and subcortical Lewy bodies (LBs); often coexists with AD	Abnormal tau and TDP-43 proteins in the frontal and temporal lobes	Accumulation of α-synuclein in LBs	Specific inherited gene mutation	Accumulation of tau and amyloid plaques
Cerebral damage		Diffuse cerebral atrophy	Widespread LBpathology; variable cortical atrophy	Severe atrophy	Atrophy in subcortical regions and cortical LB pathology	Neuronal loss in caudate nucleus and putamen	Combination of AD and vascular lesions
Prominent symptoms		Memory dysfunction	Fluctuating cognition, visual hallucinations, and parkinsonism	Personality and language disturbances	Impaired attention, executive dysfunction, memory issues	Cognitive decline with behavioral disturbances	Memory loss, cognitive decline, executive dysfunction
Visuospatial abilities		Severely impaired	Markedly impaired	Preserved	Moderately impaired	Impaired	Often impaired
Language problems		Understanding; speaking	Speaking	Thinking; understanding; reading	Thinking; speaking	Speaking	Variable; may mirror AD difficulties
Mood		Depression, anxiety, suspiciousness	Depression, anxiety, apathy, confusion	Marked irritability, lack of guilt, alexithymia euphoria, apathy	Depression, anxiety, apathy	Depression, irritability, aggression, apathy	Depression, anxiety, apathy
Intellectual deficit		Yes	Yes	No	Yes	Yes	Yes
Psychotic features		Delusion of misidentification or prejudice secondary to memory impairment type	Prominent visual hallucinations, delusions	Rare persecutory delusionsand bizarre behaviors	Visual hallucinations, paranoid delusions	Psychosis	Possible delusions and hallucinations
Appetite, dietary change		Anorexia and weight loss	Weight loss	Increased appetite, carbohydrate craving 80%, weight gain	Weight loss	Weight loss	Variable (weight loss or gain)
Progression to death		11.8 ± 0.6 years	5–8 years after diagnosis	8.7 ± 1.2 years	5–10 years after onset	15–20 years after onset	Variable (faster than single dementia types)
Cause of death		Aspiration pneumonia	Aspiration pneumonia, complications of immobility, and infections	Physical changes that can cause skin, urinary tract, and/or lung infections	Complications from immobility, aspiration pneumonia	Complications from immobility, infections, aspiration pneumonia	Cardiovascular disease, pneumonia, infections

**Table 2:** MS platforms for glycolipid biomarkers. Strengths and limitations [26,27,28,29,30,31,32,33].

Platform/Workflow	Key Features	Advantages	Limitations	Typical Use Cases
LC-MS/MS with Orbitrap/QuadrupoleTime of Flight (QTOF)	Chromatographic separation High-resolution tandem MS (MS/MS)	Quantitative, robust, reduces isobaric interference Structural info on fatty acyl chains Sensitive	Requires optimized chromatography Need for derivatization or specialized columns	Discovery and validation
Shotgun Lipidomics (Direct Infusion, Orbitrap/TripleTOF)	Rapid, high-throughput profiling Direct infusion, no LC	Broad coverage Quick surveys Minimal preparation	Ion suppression Poor isomer/isobar separation Less quantitative	Initial screening before separation
MALDI-MSI	Spatial tissue mapping Moderate to high resolution	Links molecular and anatomical data Enables regional distribution analysis High sensitivity with derivatization and high-resolution analyzers	Lower quantitation than LC-MS Matrix/analyte suppression	Mapping Correlation with plaques, vessels, microinfarcts
Desorption Electrospray Ionization (DESI)–MSI and Secondary Ion Mass Spectrometry (SIMS) Imaging	Ambient ionization (DESI) Utra-high-resolution imaging (SIMS)	Minimal sample preparation (DESI) Sub-micron resolution (SIMS)	Limited mass range and fragmentation (SIMS) Complex data analysis	Subcellular mapping Complementary spatial lipidomics
IMS-MS	Separates isomers and isobars by shape/size	Resolves complex GGs High confidence in structural identification Boosts discovery	Requires specialized instruments	Biomarker discovery Detailed structural assignment
Targeted Derivatization and Glycan-Specific Workflows	Chemical modifications Specialized columns	Improves chromatographic behavior and MS sensitivity Resolves isomers	Extra sample preparation complexity	Quantifying disease-relevant isomers
Quantitative MSI and LC-MS Hybrid	Combines MSI with microextraction and LC-MS/MS	Spatial localization and quantitative data Emerging gold standard	Complex workflow	Tissue-to-histopathology mapping Anatomical and quantitative mapping

**Table 3:** Glycolipid classes relevant to mixed dementia discovered by MS-based approaches.

Glycolipid Class	Representative Species	Relevance to Mixed Dementia	Representative Citation
GGs (mono-/di-/tri-sialo)	GM1 (d18:1/18:0); GM2; GM3 (d18:1/16:0; d18:1/18:0); GD1a; GD1b; GT1b	Abundant in neuronal membranes and synapses Altered sialylation indicates membrane degradation and inflammation GM2/GM3 elevated near plaques and in white matter	[66]
Sulfatides (sulfated galactocerebrosides)	ST (d18:1/24:0)	Enriched in myelin Early loss linked to AD and vascular myelin injury Sensitive marker of demyelination	[166]
GalCer/GlcCer	GalCer (d18:1/24:0); GlcCer species	Core myelin lipids Shifts indicate demyelination in ischemic regions Reflect altered GSL metabolism	[167]
Ceramides	Cer (d18:1/16:0); Cer (d18:1/24:1)	Products of SM breakdown Elevated in neurodegeneration and vascular inflammation Promote apoptosis and Aβ production	[168]
Sphingomyelins	SM (d18:1/18:0); SM (36:1)	Structural membrane lipids SM/ceramide ratio changes reflect membrane injury Altered in mixed dementia tissue	[169]
GG degradation intermediates	GM2; lactosylceramides (LacCer)	Indicate increased glycosidase activity Reflect lysosomal/autophagy dysfunction Accumulate around plaques and ischemic zones	[66]
Glycolipid oxidized/ truncated forms	Oxidized ceramides; truncated GGs	Markers of oxidative stress and lipid peroxidation Elevated near microinfarcts and plaques	[170]

**Table 4:** Overview of GSL expression in various types of dementia.

Dementia Type	Most Promising GSL Biomarkers	Direction of Change	References
AD	GM1, GM2, GM3, GD1a, GD1b, GD2, GD3, GT1a, GT1b, GQ1b, GQ1bα, di-*O*-Ac-GT1a, *O*-Ac-GD1b, ShexCer, GalNAc-GD1a, GGs (d18:1)	↑ GM1 (d18:1/18:0), GM2, GM3, GD2, GD3, GT1a, GQ1b, GQ1bα, di-*O*-Ac-GT1a, *O*-Ac-GD1b, GGs (d18:1), TG, CE, PE ↓ GD1a, GD1b, GT1b, ShexCer, GalNAc-GD1a	[42,45,51,65,66,70,71,121,122,136,137,138,171]
DLB	GM1 and GD1a, Cer (d18:1/18:0), GlcCer (d18:1/18:0), SphM (d18:1/18:0), GlcSph (d18:1), GalSph (d18:1)	↑ GM2, GM3 ↓ GM1 and GD1a, GalSph and Cer vs. controls/PD	[98]
PDD	Cer 24:1, 14:0, 18:0, 20:0	↓ Cer 24:1, 14:0 ↑ Cer 18:0, 20:0	[122]
FTD	GM1, GM2, GM3, GD1, GD2, GD3, GT1a, GalNAc-GD1a, HexCers (22:0 GlcCer, GalCer)	↑ GM1, GM2, GM3GD1, GD2, GD3, GT1a, GT3, GQ1c ↓GalNAc-GD1a, HexCers (22:0 GlcCer, GalCer)	[120,121,122]
HD	Ceramides (chain length shift), SM, GM1	Loss of very-long-chain Cer/SM Enrichment inlong-chain Cer/SM ↓ GM1, GM2, GD1a, and GD1b; improvement with exogenous GM1	[134,136,138]
Mixed dementia	GM2, GM3, Cer (d16:1/24:0), Cer (d18:1/16:0), Hex2Cer (d16:1/16:0), HexCer (d18:1/18:0), SM (d16:1/16:0, 20:0), SM (d18:2/22:0), GlcCer, GalCer, sphingolipids (d16:1, d18:1)	↑ GM2, GM3 (in/around Aβ plaques) ↑GM3 and membrane breakdown markers in mixed dementia ↓Sphingolipid d16:1 (VD), GM1, GD1a, and GT1b ↑Sphingolipid d18:1 (AD)	[66,162,165]

## Data Availability

No new data were created or analyzed in this study. Data sharing is not applicable to this article.

## Abbreviations

The following abbreviations are used in this manuscript:

AD	Alzheimer’s disease
AI	artificial intelligence
APP	amyloid precursor protein
Aβ	amyloid-β peptide
BBB	blood–brain barrier
BMP	bis(monoacylglycero)phosphate
bvFTD	behavioral variant FTD
CE	cholesteryl ester
CSF	cerebrospinal fluid
DAT-SPECT	dopamine transporter–single-photon emission computed tomography
DESI	desorption electrospray ionization
DLB	dementia with Lewy body
ELISA	enzyme-linked immunosorbent assay
ESI	electrospray ionization
FDG-PET	fluoro-deoxyglucose positron emission tomography
FTD	frontotemporal dementia
GalCer	galactosylceramide
GalSph	galactosylsphingosine
GCase	β-glucocerebrosidase
GG	ganglioside
GlcCer	glucosylceramide
GlcSph	glucosylsphingosine
*GRN*	progranulin gene
GroPIn	glycerophosphoinositol
GSL	glycosphingolipid
HD	Huntington’s disease
HDD	Huntington’s disease dementia
Hex1Cers	monohexosylceramides
*HEXA*	hexosaminidase subunit alpha gene
HexCers	hexosylceramides
*HTT*	huntingtin gene
IMS	ion mobility spectrometry
LacCer	lactosylceramide
LB	Lewy body
LBD	Lewy body dementia
LC-MS	liquid chromatography coupled with mass spectrometry
MALDI	matrix-assisted laser desorption/ionization
mHTT	mutant huntingtin
MRI	magnetic resonance imaging
ML	machine learning
MS	mass spectrometry
MS/MS	tandem mass spectrometry
MSI	mass spectrometry imaging
NfL	neurofilament light chain
NFT	neurofibrillary tangle
PC	phosphatidylcholine
PD	Parkinson’s disease
PDD	Parkinson’s disease dementia
PE	phosphatidylethanolamine
PET	positron emission tomography
PG	phosphatidylglycerol
PGRN	progranulin
PI	phosphatidylinositol
PS	phosphatidylserine
p-tau	phosphorylated tau
PTM	post-translational modification
QTOF	quadrupole time of flight
SHexCer	sulfatide
SIMS	secondary ion mass spectrometry
SM	sphingomyelin
SP	senile plaque
TG	triacylglycerol
TLC	thin-layer chromatography
TMM	transgenic mouse model
UHPLC MS/MS	ultra-high-performance liquid chromatography coupled to tandem MS
VD	vascular dementia
VLCFA	very-long-chain fatty acid
